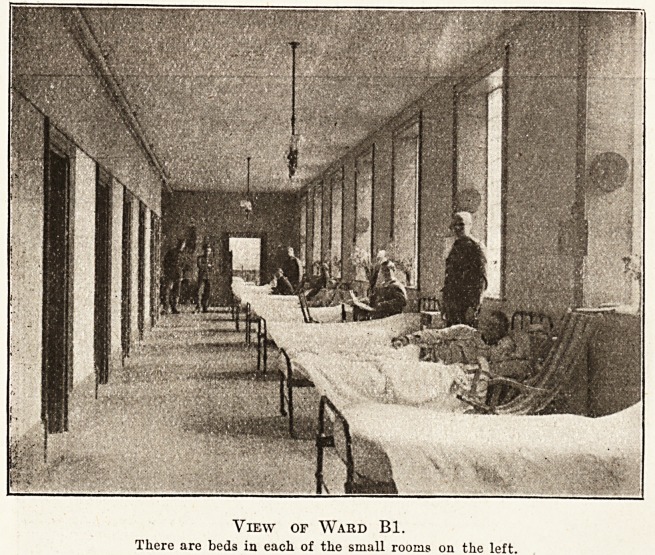# The Guiding Principles of the Scheme

**Published:** 1914-10-10

**Authors:** 


### TERRITORIAL HOSPITALS.*

### THE GUIDING PRINCIPLES OF THE SCHEME.

The hospital provision for the reception of the wounded from the war, so far as the Army is concerned, is entirely in the hands of the War Office. They rely upon three groups of hospitals which collectively could, if required, provide a sum total of 50,000 beds. The three groups are, first, the military Hospitals which could provide if urgently necessary, for 20,000 wounded; secondly, the Territorial hospitals, which have provision for nearly 12,000 beds, which can be increased by tents and other extensions to 20,000; and thirdly, the voluntary hospitals, which it is estimated will collectively provide 10,000 beds. Fifty thousand beds are considered by the War Office to be ample, and possibly more than ample, to meet all the requirements of the wounded who are likely to reach our shores. This view was expressed before the wounded had begun to arrive, and is confirmed by the experiences of the last month, which shows that the great majority of the soldiers admitted to the hospitals in England from the Front have recovered sufficiently within eight days to a fortnight to enable their wounds to heal so that they can be sent to their friends, or for change elsewhere on furlough for a brief space before they rejoin the Colours at the Front.

Principal Front of 5th Northern General Hospital, Leicester.

The organisation throughout the United Kingdom for the reception of the sick and wounded from the war originated with Sir Alfred Keogh when Medical Director-General of the Army Medical Service. In 1907 he evolved a practical and extended plan which has steadily been developed. Under his direction steps were taken (*a*) for the establishment in selected buildings throughout the country of twenty-three general hospitals containing a minimum of 500 beds, (*b*) to set up and to make provision for an adequate medical and nursing staff locally for each such hospital. Many of the buildings selected had ample space surrounding them, so that in some cases it might be possible to provide in the adjacent grounds further hospital accommodation for from 1,000 to 1,500 extra patients. The selection of suitable buildings, mainly public buildings, was decided upon in substitution for the tent hospitals originally contemplated. The organisation of these hospitals has been carried out by the Royal Army Medical Corps, and also their equipment. For a list of these hospitals see The Hospital, August 15 last (*vide* footnote).

It reflects great credit on the department of the Medical Director-General of the Army, especially in view of the attempts which are being made to raise the price of bedsteads and other necessary articles of hospital equipment, that most things necessary to furnish and open each of these hospitals were purchased and stored in sections, so that it has been relatively easy to transfer each complete equipment from the stores to the Territorial hospital to which it was registered and assigned. Attached to each hospital there is an adequate ambulance service, so that the sick and wounded may be readily conveyed to the hospitals, and, as they convalesce, from them to the semi-convalescent institutions provided in connection with every base hospital for the accommodation of the relatively few cases which may need such care.

Originally, at a number of the twenty - three Territorial hospitals it was arranged to commandeer school buildings, technical and art schools, and other groups of buildings chiefly owned by local authorities, which it was considered might in case of emergency be converted for use as a portion of the Territorial hospital unit. These arrangements were provisionally made seven years ago, and when war was declared and notice was given to the authorities concerned that the groups of municipal and other public buildings originally named were required for hospital purposes, strong remonstrance was made by representatives of the residents and ratepayers on the ground that the education of the children and the provision made for the ordinary work of the cities and towns where these buildings were situated would be so grievously interfered with, if not

* For list see The Hospital, August 15, 1914, page 553, and August 22, 1914, page 574.

entirely stopped, as to render it undesirable that they should be commandeered. It was further pointed out that many of the buildings, schools especially, were not suitable for hospital purposes, and before they could be used as part of a Territorial hospital unit they must undergo considerable structural alterations which would involve much expense. These objections and other causes led to a reconsideration of the original proposals, and a scheme of tent hospitals was gone carefully into, with the result that it was negatived as unsuitable, having regard to the fact that the hospitals would probably be required for an entire year or longer.

### Temporary Hospitals.

The need for extra hospital provision being, however, paramount, accommodation was made effectively. Temporary hospitals were provided (1) in existing buildings like those originally named, (2) in portions of the colleges connected with Universities, (3) in old buildings like the late County Asylum near Leicester, and (4) in hut hospitals specially erected for the purpose. In several cases it is contemplated, should necessity arise, to increase the number of beds (520) of several of the Territorial hospitals by the erection of huts or tents as circumstances may render best, which additions will be administered from the central buildings which have been already prepared and many of which are already occupied by men wounded in the war. We have thought it would prove interesting to give plans and particulars of at least one of the Territorial hospitals, because they represent a new feature in British hospitals and have been called into existence by and will remain in use during the continuance of the Great War.

View of Ward B1.
There are beds in each of the small rooms on the left.

The organisation of these Territorial hospitals has sprung into existence so rapidly and may continue so long that we can hardly realise now the importance of preserving a record to refer to later on. Yet it must not be forgotten that these institutions sooner or later are destined to disappear as rapidly as they arose, and can necessarily leave little to recall their value beyond the memories of those who were personally engaged in working them.

We have, therefore, much pleasure in publishing a descriptive account of the Territorial Hospital at Leicester, which has been organised, equipped and occupied with remarkable speed and excellent results, thanks to the energy and united efforts of the people of Leicestershire and the town of Leicester. It is a most amazing fact that so alert, loyal, whole-hearted, and able a population as that of the town of Leicester should rest content to have associated with them as their member of Parliament a man who stands almost alone in these islands, as the *Times* of the 1st instant pointed out in a leading article "Helping the Enemy," and is so not entitled to enjoy the "contemptuous indifference" of the public, much less of the electorate of Leicester. "Though his influence at home is nil, his utterances are materially helping the enemy abroad, where he is still supposed to represent the Labour Party." The *Times* concludes, "We do not suppose that Mr. Macdonald wishes to help the enemy, but if he does not it must be painful to him to realise that no paid agent of Germany has served her better than himself." We call attention to this matter here because the writer, as a Leicestershire man, desires that the town of Leicester should put itself right with the country and the Empire by exhibiting the same vigorous spirit in upholding the honour of its citizens as loyal Englishmen, as it has done in every other matter which can in any way help to bring victory to our nation, in the just cause for which our soldiers and sailors are giving their lives in certainly the most memorable war that the world has ever known.

As we still turn with interest to the life of Florence Nightingale for her accounts of the Crimean hospitals, so we may well make a point of preserving a vivid picture of a typical base hospital at work to-day. The flood of literature occasioned by the war has, it is curious to note, almost completely passed by the vital feature of it represented by our Territorial hospitals. Ephemeral points at home and abroad have been relentlessly exploited, but an intelligent account of these institutions has not been readily available.

## Figures and Tables

**Figure f1:**
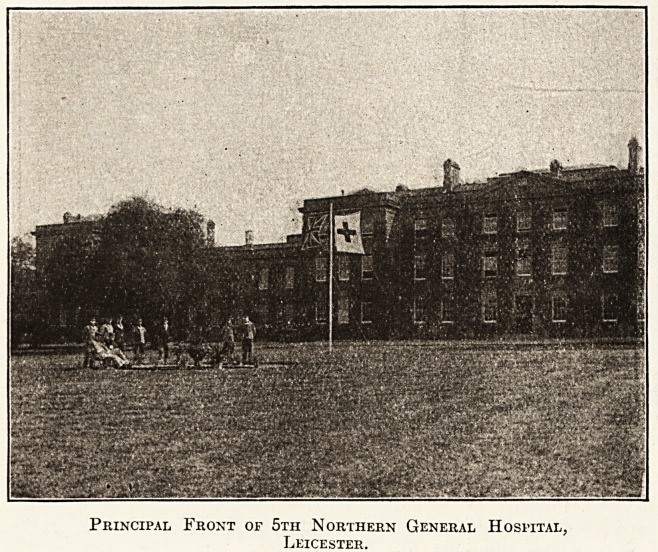


**Figure f2:**